# The role of SWI/SNF chromatin remodelers in the repair of DNA double strand breaks and cancer therapy

**DOI:** 10.3389/fcell.2022.1071786

**Published:** 2022-12-20

**Authors:** Maria Sadek, Anand Sheth, Grant Zimmerman, Emily Hays, Renier Vélez-Cruz

**Affiliations:** ^1^ Biomedical Sciences Program, College of Graduate Studies, Midwestern University, Downers Grove, IL, United States; ^2^ Chicago College of Osteopathic Medicine, Midwestern University, Downers Grove, IL, United States; ^3^ Department of Biochemistry and Molecular Genetics, College of Graduate Studies, Midwestern University, Downers Grove, IL, United States; ^4^ Chicago College of Optometry, Midwestern University, Downers Grove, IL, United States; ^5^ Chicago College of Pharmacy, Midwestern University, Downers Grove, IL, United States

**Keywords:** chromatin remodelers, homologous recombination, double strand break repair, cancer therapy, DNA end resection, SWI/SNF, PARP inhibitors

## Abstract

Switch/Sucrose non-fermenting (SWI/SNF) chromatin remodelers hydrolyze ATP to push and slide nucleosomes along the DNA thus modulating access to various genomic loci. These complexes are the most frequently mutated epigenetic regulators in human cancers. SWI/SNF complexes are well known for their function in transcription regulation, but more recent work has uncovered a role for these complexes in the repair of DNA double strand breaks (DSBs). As radiotherapy and most chemotherapeutic agents kill cancer cells by inducing double strand breaks, by identifying a role for these complexes in double strand break repair we are also identifying a DNA repair vulnerability that can be exploited therapeutically in the treatment of SWI/SNF-mutated cancers. In this review we summarize work describing the function of various SWI/SNF subunits in the repair of double strand breaks with a focus on homologous recombination repair and discuss the implication for the treatment of cancers with SWI/SNF mutations.

## Introduction

### SWI/SNF chromatin remodelers

**FIGURE 1:**
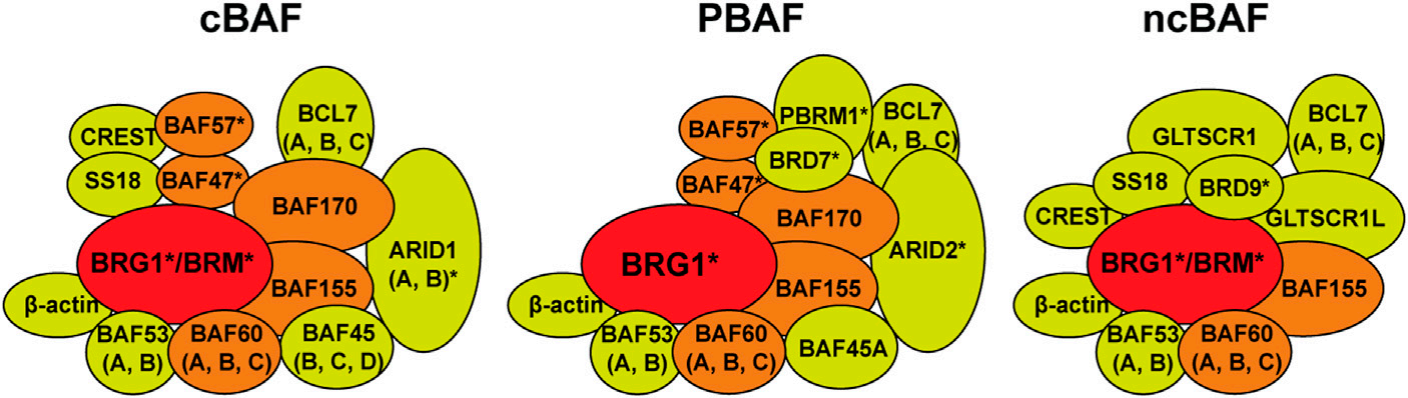
Switch/Sucrose non-fermenting (SWI/SNF) chromatin remodelers. There are three forms of the SWI/SNF chromatin remodelers in somatic mammalian cells. The canonical BAF (cBAF, BRG1-associated factors), the polybromodomain BAF (PBAF), and the non-canonical BAF (ncBAF). These complexes can contain either the BRG1 or BRM ATPase (red). cBAF and PBAF complexes contain a group of core subunits (BAF47, BAF57, BAF60, BAF155, BAF170, orange) and a number of accessory factors of unknown function (yellow). The ncBAF only contains BAF60 and BAF155 core subunits. PBAF contains the polybromodomain protein PBRM1 (BAF180) and BRD7, while ncBAF contains BRD9. Subunits marked with an asterisk (*) are known to be mutated in a variety of cancers.

Switch/Sucrose non-fermenting (SWI/SNF) chromatin remodelers are a group of large macromolecular complexes first identified in yeast as transcription regulators required for the expression of genes related to the mating-type switch locus and sucrose fermentation (Neigeborn and Carlson, 1984; Stern et al., 1984). These complexes hydrolyze ATP in order to push and slide nucleosomes and thus modulate access to certain DNA loci for transcription. In general, somatic mammalian cells have three distinct SWI/SNF chromatin remodeler complexes. These complexes are composed of 10–15 subunits, some of which are shared, and some that are exclusive to each complex (Phelan et al., 1999; Mashtalir et al., 2018; Michel et al., 2018). All complexes contain one ATPase, either BRG1 or BRM, and this is the only catalytic activity within the complex. These complexes are known as the canonical BAF (cBAF, canonical BRG1-associated factor), the non-canonical BAF (ncBAF), and the polybromo-associated BAF (PBAF) (Mashtalir et al., 2018) (Figure 1). These complexes also contain a group of core subunits (BAF47, BAF57, BAF60, BAF155, BAF170) that help with complex assembly and stability, particularly the stability of the ATPase, and also stimulate the nucleosome remodeling capacity of these complexes (Phelan et al., 1999; Pulice and Kadoch, 2016; Mashtalir et al., 2018). Finally, these complexes contain several accessory subunits that are of yet unknown function, and it is unclear whether or to what extent these accessory subunits affect the chromatin remodeling capacity of these complexes, the recruitment of these complexes to a particular genomic locus, or the integrity of the complex. It is important to note that there is another group of SWI/SNF complexes that are exclusively expressed in in particular cell types only during development (pluripotent embryonic stem cells, neural stem cells, post-mitotic neurons, *etc.*) and these complexes will not be discussed in this article but have been discussed elsewhere (Ho et al., 2009; Kadoch and Crabtree, 2015).

**FIGURE 2:**
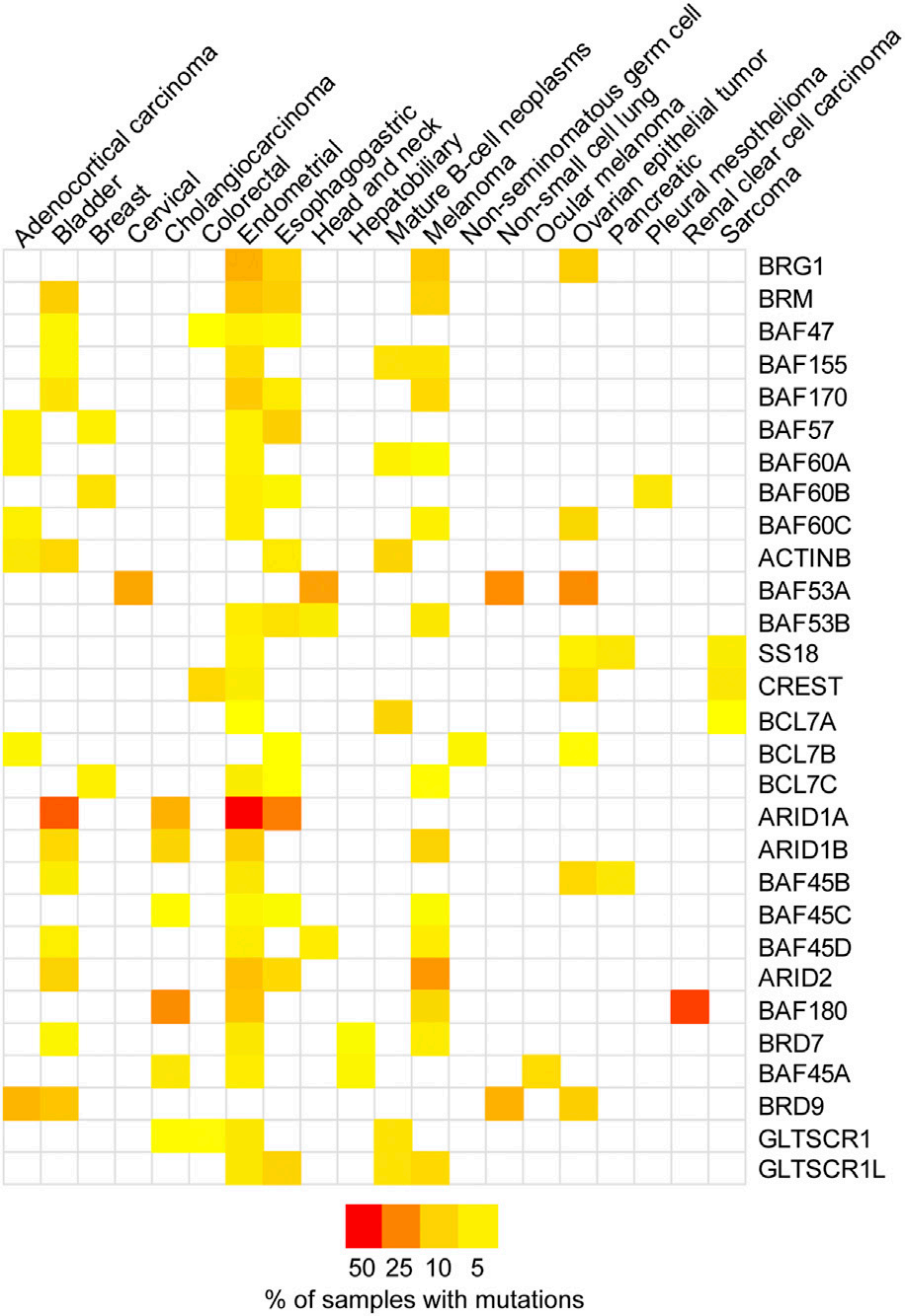
SWI/SNF subunits are mutated in cancer. A heatmap representing the mutation frequency for individual SWI/SNF subunits across different cancer types. The data was obtained from The Cancer Genome Atlas (TCGA Pan-Cancer Atlas study, *n* = 10,967 samples) through cBioPortal (Cerami et al., 2012; Gao et al., 2013). The top four cancer types with a mutation frequency higher than 2% were selected for each subunit. Supplementary Table S1 provides the mutation frequencies.

SWI/SNF complexes have a strong link to cancer. The first example of the link between SWI/SNF chromatin remodelers and cancer development was the fact that 100% of malignant rhabdoid tumors (MRTs), a particularly aggressive type of childhood cancer, were found to carry mutations in the *SMARCB1* gene (BAF47 protein) (Versteege et al., 1998). This discovery drew attention to these complexes and later proteomic and bioinformatic analyses showed that multiple subunits within these complexes are mutated at very high frequencies in up to 20% of human cancers (Kadoch et al., 2013). Indeed, SWI/SNF chromatin remodelers are the most highly mutated epigenetic regulators in human cancers. While the types of mutations varied, in general these mutations resulted in the loss of expression of the given subunit. Both ATPases, BRG1 and BRM (*SMARCA4* and *SMARCA2* genes, respectively) were among the highly mutated subunits, but not the only ones. Using data from The Cancer Genome Atlas (TCGA) we compiled the top four cancer types with SWI/SNF subunit mutations (Figure 2; Supplementary Table S1) (Cerami et al., 2012; Gao et al., 2013). These complexes also contain proteins containing AT-rich interacting domain (ARID) and these subunits are also frequently mutated (Figure 2) (Kadoch et al., 2013). ARID1A/B are mutually exclusive components of the cBAF complex and ARID2 is an exclusive component of the PBAF complex (Figure 1) (Mashtalir et al., 2018). These ARID domain-containing proteins are of interest because they can interact directly with DNA and are thought to be important for the recruitment of the SWI/SNF complexes to various genomic loci. Another group of accessory factors mutated at high frequencies in cancer are bromodomain-containing proteins BRD7, BRD9, and PBRM1 (Kadoch et al., 2013). These proteins, through their bromodomains, have the capacity to bind to acetylated proteins and are thought to be important for binding to acetylated histones and thus targeting SWI/SNF complexes to particular genomic regions.

SWI/SNF complexes have been studied extensively in the last 35 years and most of the studies have focused on the catalytic subunits and their role in multiple transcriptional pathways. For instance, it is well documented that BRG1 and BRM are important for the expression of cell cycle progression genes through their interaction with the retinoblastoma (RB) tumor suppressor (Dunaief et al., 1994; Strobeck et al., 2000, 2002; Weissman and Knudsen, 2009). BRG1 is also important for driving the expression of *Myc* in certain leukemias (Shi et al., 2013). These ATPases are also known to be important for the transcriptional programs that direct cellular differentiation (Flowers et al., 2010). There are some studies that have also investigated the importance of subunits other than the ATPases, such as the ARID1A, ARID1B, and ARID2, and their importance for these transcriptional programs related to cell cycle progression and cell differentiation (Nagl et al., 2005; Xu et al., 2012; Sun et al., 2016; Mathur et al., 2017; Wang et al., 2020). There is, however, a lack of understanding of the function of the rest of the SWI/SNF accessory subunits in terms of transcription (or any other function) in the context of their tumor suppressor capacity.

In addition to their role in transcription, more recent work has implicated SWI/SNF complexes in the preservation of genomic integrity. It would be rational to think that if SWI/SNF complexes modulate access to DNA during transcription, these complexes would also be required for any other process that requires access to DNA, such as DNA repair. More importantly, given the fact that cancer treatments such as radiotherapy and most chemotherapeutic agents kill cancer cells by damaging DNA, if these complexes are important for DNA repair, then SWI/SNF-mutated cancers would also have a DNA repair vulnerability that can potentially be exploited therapeutically. In this review we will focus on the recent work that has been done to define the role of SWI/SNF chromatin remodeling complexes in DNA repair, the function ascribed to various SWI/SNF subunits, and the potential implications for the treatment of cancers bearing mutations in SWI/SNF subunits.

### DNA double strand break repair

DNA double strand breaks (DSBs) are among the most toxic types of DNA damage, as they can result in chromosomal aberrations, in addition to insertions and deletions and many other mutagenic outcomes (Stinson and Loparo, 2021). In human cells there are two major pathways that repair DSBs. The non-homologous end joining (NHEJ) pathway is the main repair mechanism that deals with DSBs in human cells, and it mainly consists of the joining of DNA ends with no regard for sequence homology (Davis and Chen, 2013; Stinson and Loparo, 2021). This pathway is highly efficient and quick, but it can lead to the generation of insertion/deletions (indels) and in the presence of multiple DSBs can lead to the generation of chromosomal aberrations such as translocations, which themselves can be carcinogenic. The second pathway that repairs DSBs in human cells is homologous recombination (HR). HR is a more complex pathway that uses a sister chromatid as a template for accurate repair of DSBs (Ranjha et al., 2018). Due to the requirement of a sister chromatid for HR, it can only take place after DNA replication (late S and G2) and thus it is less frequently used.

During HR the DSB is recognized by the MRN complex (MRE11-RAD50-NBS1) and the ATM kinase (Lee and Paull, 2005; Paull and Lee, 2005). The MRN complex recruits the CtIP nuclease to the break and this endonuclease cleaves the DNA backbone near the 5′ end (Sartori et al., 2007). This process of trimming the 5′ end of the DNA is known as DNA end resection and results in the generation of stretches of ssDNA with a free 3′-OH terminal that will become coated by the ssDNA binding protein RPA. These ssDNA regions coated by RPA activate the ATR kinase, which will then phosphorylate RPA itself, the CHEK1 kinase, and other target proteins as part of the DNA damage response (DDR) (Zou and Elledge, 2003; Cimprich and Cortez, 2008; Duursma et al., 2013). The breast cancer susceptibility gene 1 (BRCA1) is known to stimulate the resection process mediated by CtIP (Cruz-García et al., 2014). While DNA end resection is initiated by MRE11 and CtIP, extensive resection is conducted by the EXO1 and DNA2 exonucleases with the help of other factors (Cejka, 2015; Ranjha et al., 2018). These resected ends coated by RPA will become coated by the RAD51 recombinase, which will then mediate the strand invasion and homology search steps of HR. The replacement of RPA for RAD51 is mediated by BRCA2 (Roy et al., 2011). After the strand invasion and homology search has been completed, DNA polymerases extend the 3′OH ends through the region where the break occurred, which leads to the formation of a double Holliday junction (dHJ) intermediate (Ranjha et al., 2018). Double Holliday junctions can be further processed through a dissolution mechanism that requires BLM-TOPO3α-RMI1/2 complex which prevents the formation of crossovers and maintains genome integrity. Alternatively, dHJ can be processed by a resolution mechanism that employs nucleases that cleave this DNA structure at different sites to resolve this repair intermediate (Ranjha et al., 2018). Resolution of the dHJ can generate crossover products, which are mutagenic. Because HR uses a sister chromatid, which is an identical copy of itself, as a template to repair the DSB, this pathway is more accurate than the NHEJ pathway.

Cells with defects in HR or NHEJ are sensitive to DNA damaging agents that cause DSBs, which is the case for the vast majority of anti-cancer chemotherapeutics and radiation therapy (Jackson and Helleday, 2016; Nickoloff et al., 2017; Hopkins et al., 2022). Moreover, cells that are deficient in HR specifically are sensitive to poly (ADP-ribose) polymerase inhibitors (PARPi). While the mechanism by which PARPi kill HR-deficient cells is still being debated, it is thought that these compounds inhibit PARP1 at DSBs and this enzyme becomes trapped at these breaks in a way that can only be repaired through HR and thus HR-deficient cells die upon treatment with these compounds. PARPi are used for the treatment of ovarian cancers with *BRCA1* or *BRCA2* mutations and there are clinical trials assessing their efficacy in other HR-deficient cancer types (Helleday, 2010; Rouleau et al., 2010; Dréan et al., 2016; D’Andrea, 2018; Cong and Cantor, 2022).

While NHEJ does not seem to be particularly sensitive to the chromatin environment of the DSB in order for it to work, HR seems to be quite sensitive to the chromatin state (Miller et al., 2010; Chen et al., 2019). Thus, there is a direct link between HR efficiency and chromatin environment, which is why in this review we focus on HR and the role of SWI/SNF chromatin remodelers.

### SWI/SNF ATPases: BRG1 and BRM

**FIGURE 3:**
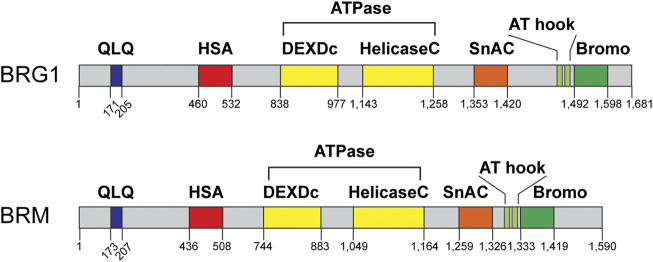
BRG1 and BRM domain structures. BRG1 and BRM are the sole catalytic subunits within these complexes and are mutually exclusive within the complex. Each ATPase contains a QLQ protein interaction domain (QLQ, blue), a helicase SANT-associated domain (HSA, red), the ATPase/helicase domain (yellow), a SNF2 ATP-coupling (SnAC, orange) domain, two A-T hook motifs (light green), and a bromodomain (Bromo, green). Domain structure information obtained from NCBI and cBioPortal.

The SWI/SNF ATPases BRG1 and BRM have garnered most of the research interest regarding the role of these complexes in DNA repair, as these proteins are the only catalytic subunits within these complexes (Phelan et al., 1999; Pulice and Kadoch, 2016; Mashtalir et al., 2018). At the cellular level, both of these ATPases can be downregulated or inactivated, but in mice, inactivation of BRG1 results in embryonic lethality, while inactivation of BRM results in mice that are normal, albeit ∼15% heavier (Reyes et al., 1998; Bultman et al., 2000). It is important to note, however, that tissue-specific inactivation of BRG1 in mice testes results in infertile mice due to a defect in meiotic recombination, which is similar to homologous recombination repair (Kim et al., 2012; Wang et al., 2012). In terms of protein structure, BRG1 and BRM share a high degree of sequence identity. Both ATPases have QLQ domains thought to be important for protein-protein interactions, a helicase SANT-associated (HSA) domain, thought to be important for the binding of actin-related proteins (ARPs), ARID1A, and DNA binding, an ATPase domain that hydrolyzes ATP, an RB-binding region, a SNF2-ATP coupling (SnAC) domain, two A-T hook motifs, and a bromodomain that binds to acetylated proteins (Figure 3) (Chen and Archer, 2005; Trotter et al., 2007; Sen et al., 2013; Hota and Bruneau, 2016; Clapier et al., 2017; Mashtalir et al., 2018). While multiple studies have investigated the function of some of these domains in the context of transcription, it is less clear what the function of these domains is in the context of DNA repair.

The recruitment of SWI/SNF chromatin remodelers to particular genomic loci largely depends on the specific genomic site. These complexes have been shown to interact with and be recruited by nuclear receptors and transcription factors in the context of transcription regulation (Swinstead et al., 2016). Pioneer transcription factors are known to bind genomic loci in the absence of chromatin remodelers but have been shown to be helped by SWI/SNF complexes (Swinstead et al., 2016). In the context of DNA repair, SWI/SNF complexes are recruited by transcription regulators such as the retinoblastoma protein, the ATR kinase, PARP1, bromodomain-containing proteins, and many other mechanisms (Gong et al., 2015; Shen et al., 2015; Velez-Cruz et al., 2016; Chen et al., 2019). It is likely that the recruitment of SWI/SNF complexes to DSBs is context-dependent and that the chromatin environment dictates whether these complexes are needed. Multiple studies have shown that BRG1 and BRM are both recruited to DSBs, but there are various models for the function of BRG1 at DSBs and the effect of the inactivation of BRG1 in terms of the DNA damage response, while the role of BRM is unclear (Gong et al., 2015; Qi et al., 2015; Velez-Cruz et al., 2016; Wiest et al., 2017; Chen et al., 2019; Hays et al., 2020).

### The role of BRG1 in the DNA damage response (DDR)

Initial studies showed that inactivation of BRG1 results in a defect in the phosphorylation of histone H2AX (γH2AX), maker for DSBs, and sensitivity to DSB-inducing agents (Park et al., 2006). These studies, however, used the overexpression of a dominant negative version of BRG1 and not the downregulation of the gene by siRNA or shRNA in most of their assays (Park et al., 2006). The same group later showed that BRG1, through its bromodomain, interacts with acetylated γH2AX, that this interaction is important for the repair of DSBs, and that the histone acetylation is carried out by the GCN5 histone acetyltransferase (Lee et al., 2010). They proposed that BRG1 can be recruited to γH2AX nucleosomes through interactions between acetylated histones and the BRG1 bromodomain and that this activation loop was responsible for the spreading of BRG1 along the DSB region (Lee et al., 2010). Indeed, this group also showed that overexpression of the BRG1 bromodomain dimer alone has a dominant negative effect by displacing endogenous BRG1 from chromatin. The overexpression of this bromodomain dimer sensitizes cells to DNA damage, impairs γH2AX and 53BP1 foci formation, impairs the G2/M checkpoint activation, and impairs the repair of DSBs (Kwon et al., 2015a). Another study showed that BRG1 is a substrate for ATM and that phosphorylated BRG1 (at S721) is recruited to γH2AX sites and that this phosphorylation event is important for the repair of DSBs (Kwon et al., 2015b). Some of these earlier studies regarding the activation of the ATM kinase and γH2AX foci are in conflict with more recent work from other groups. For instance, the Bultman lab showed that simultaneous downregulation of both ATPases (BRG1 and BRM) using shRNA, impairs the activation of the ATR kinase, but not the ATM kinase, with the exception of γH2AX induction after treatment with the topoisomerase II poison etoposide, which was reduced in cells lacking both ATPases (Smith-Roe et al., 2015). Our laboratory, using shRNA against BRG1 or inactivating BRG1 using CRISPR/Cas9 targeting the *SMARC4* gene (gene that encodes BRG1 in humans), showed increased levels of γH2AX in cells lacking BRG1 upon ionizing radiation (IR), camptothecin (CPT, a topoisomerase I poison), or bleomycin (Bleo, a radiomimetic drug), thus suggesting an intact activation of the ATM kinase. We also observed no difference in phosphorylated ATM (pATM) foci after bleomycin treatment between control cells and cells lacking BRG1. We did detect a defect in the activation of the ATR kinase in cells lacking BRG1 upon CPT treatment or IR, similar to what the Bultman lab showed for cells lacking both ATPases (Smith-Roe et al., 2015; Hays et al., 2020). Inactivation of BRG1 in mice testes resulted in increased spermatocyte cell death, but normal levels of γH2AX that was sustained suggesting a defect in meiotic recombination (Kim et al., 2012; Wang et al., 2012). All these studies clearly establish that inactivation of BRG1 results in a DSB repair defect and in sensitivity to various forms of DNA damage, specifically those that induce DSBs (Park et al., 2006; Lee et al., 2010; Kwon et al., 2015b; 2015a; Smith-Roe et al., 2015; Hays et al., 2020).

### The role of BRG1 in DSB repair

The earlier previously discussed studies did not address which DSB repair pathway was affected by the inactivation of the BRG1 ATPase or the mechanisms by which BRG1 promotes the repair of DSBs. A 2015 study by Qi, W. et al. showed that inactivation of BRG1 impairs HR but has no effect in NHEJ (Qi et al., 2015). They showed that BRG1 interacts with the RAD52 protein and proposed that BRG1 stimulates the replacement of RPA bound to ssDNA after DNA end resection for the RAD51 recombinase (Qi et al., 2015). They used a system based on SW13 human adrenal adenocarcinoma cells that do not express either BRG1 or BRM and transfected those cells with wild type BRG1 in their experiments. In some experiments, they also used U2OS cells transfected with siRNA targeting BRG1 and obtained similar results. Using these systems, they showed that the absence of both BRG1 and BRM results in reduced RAD51 foci formation and sustained RPA foci after DNA damage. They also showed that cells lacking BRG1 display lower levels of chromatin-bound RAD51 and RAD52. An experiment of interest in this study is the fact that introduction of an ATPase-dead BRG1 mutant (K798R) did correct this defect in the sustained RPA foci and reduced RAD51 foci, thus showing that BRG1 may be mediating this function in HR through protein-protein interactions but its catalytic activity is dispensable. A technical caveat with these studies is that certain cancer cells that lack BRG1 expression often undergo cell cycle arrest upon reintroduction of wild-type BRG1, and the authors did not show or comment on any cell cycle effects that the re-introduction of BRG1 may have caused (Dunaief et al., 1994; Wong et al., 2000). Also, in this case, these SW13 cells are deficient on both ATPases, which may show a different phenotype than when a single ATPase is absent, moreover upon transfection of wild-type human BRG1, those cells are still lacking BRM. There is another study that supports this model for the function of BRG1, but it relates to ARID2 (BAF200) and will be discussed in the next section (Castro et al., 2017).

A second model describing the function of BRG1 at DSBs and its function in HR proposes that BRG1 stimulates DNA end resection (Shen et al., 2015; Velez-Cruz et al., 2016; Chen et al., 2019; Manickavinayaham et al., 2019; Hays et al., 2020). We previously identified a complex containing TopBP1-E2F1-RB and this complex recruits BRG1 to DSBs (Velez-Cruz et al., 2016). Inactivation of RB results in the destabilization of the complex and the recruitment of BRG1 to DSBs is also abolished. Thus, inactivation of RB results in a defect in DNA end resection and HR (Velez-Cruz et al., 2016). More recently, we confirmed these previous findings and showed that inactivation of BRG1 itself results in a DSB repair defect through comet assays, impaired activation of the ATR kinase after DNA damage and a defect in DNA end resection and HR. We measured DNA end resection using the BrdU incorporation method, RPA foci-formation, and measured chromatin-bound RPA after DNA damage through flow cytometry, and all showed a defect in DNA end resection (Hays et al., 2020). Moreover, we showed that BRG1 promotes the recruitment of the CtIP nuclease to DSBs through laser micro irradiation experiments (Hays et al., 2020). It is important to note that we observed normal activation of the ATM kinase in our study through CHEK2 phosphorylation after IR, pATM foci formation after bleomycin treatment, and increased γH2AX foci after camptothecin or bleomycin treatments. Regarding chromatin remodeling at DSBs, using the I-PpoI nuclease system, we observed that in control cells there is a reduction in nucleosome density at the site of the break, while cells lacking BRG1 show an increase in nucleosome density at DSBs. We propose that this reduction in nucleosome density at the site of the break stimulates or stabilizes the recruitment of the CtIP nuclease, and thus DNA end resection (Hays et al., 2020). We, however, did not prove whether the ATPase activity of BRG1 was required for the recruitment of CtIP or for the reduction in nucleosome density at DSBs (Hays et al., 2020). The importance of SWI/SNF for the activation of ATR has been shown by various groups, including ours (Shen et al., 2015; Smith-Roe et al., 2015; Velez-Cruz et al., 2016; Hays et al., 2020). Similarly, the reduction in nucleosome density at DSBs upon the induction of a DSB and the increase in nucleosome density at the break in the absence of SWI/SNF has also been previously shown by multiple groups in yeast and human cells, including us (Shen et al., 2015; Velez-Cruz et al., 2016; Wiest et al., 2017; Chen et al., 2019; Hays et al., 2020). Indeed, Chen et al. designed a new reporter system to measure NHEJ and HR at the same locus and using this reporter at different genomic loci and in different cell lines showed that NHEJ does not require a reduction in nucleosome density for efficient repair, but HR does (Chen et al., 2019). They also showed that cells lacking BRG1 did not show a reduction in nucleosome density at DSBs and PARP1 was also required for this reduction. In their proposed model, PARP1 is recruited to DSBs and it recruits the histone deacetylase (HDAC) SIRT1 and the BRG1 ATPase, then SIRT1 deacetylates BRG1 on residues K1029 and K1033, thus activating its ATPase activity and reducing nucleosome density at DSBs for efficient HR (Chen et al., 2019).

This model of SWI/SNF complexes stimulating DNA end resection has also been shown in yeast (Wiest et al., 2017). The Osley lab showed that inactivation of the yeast SWI/SNF subunit Snf5 (yeast homolog of the human BAF47), which is required for the function of these complexes in yeast, results in a delayed initiation of DNA end resection (Wiest et al., 2017). They also showed a defect in the recruitment of the MRX complex (yeast homolog of the human MRN complex), a defect in the activation of the Mec1 kinase (yeast homolog of the human ATR), and a defect in the eviction of nucleosomes at the site of the break in Snf5-deleted cells. Thus, in yeast SWI/SNF complexes are also important for the removal of nucleosomes for efficient DNA end resection and HR (Wiest et al., 2017). The idea that nucleosomes could block resection has been around for a long time (Adkins et al., 2013; Chen and Symington, 2013). Methods to study DNA end resection have greatly improved over the last 10 years, particularly in yeast, where genetic manipulations are still much easier than in human cells. Multiple recent high resolution sequencing studies in yeast have shown that nucleosomes at the site of the break block resection and must be removed for efficient resection and HR (Mimitou et al., 2017; Peritore et al., 2021). Indeed, Peritore et al. recently used a strand-specific ChIP-Seq analysis in yeast to map resection products and concluded that nucleosome eviction and DNA end resection are intrinsically coupled (Peritore et al., 2021). Moreover, they showed that simultaneous inactivation of SWI/SNF and the RSC chromatin remodelers impaired both nucleosome eviction and DNA end resection (Peritore et al., 2021). Together, these studies support a role for BRG1 reducing nucleosome density at DSBs and stimulating DNA end resection.

### The role of BRM in DSB repair

The BRM ATPase is much less studied than the BRG1 ATPase. It is possible that the fact that BRG1 is more frequently mutated than BRM, plus BRG1 inactivation in mice is embryonic lethal and BRM-KO mice are healthy and fertile are some of the reasons for this lack of BRM studies (Reyes et al., 1998; Bultman et al., 2000; Kadoch et al., 2013). We recently showed using the DR-U2OS system that downregulation of BRM results a modest decrease in HR efficiency (∼15%), but the role of BRM in HR still needs to be addressed (Hays et al., 2020). It is possible that BRM acts as a backup for BRG1 in its function in HR, as, for instance, we did not find BRG1-KO cells sensitive to PARP inhibitors (PARPi), while downregulation of both ATPases simultaneously has been shown to sensitize cells to PARPi (Smith-Roe et al., 2015; Hays et al., 2020). Also, targeting BRM in non-small cell lung cancer cells that have lost BRG1 expression seems to sensitize these cells to IR (Zernickel et al., 2019). It is also clear that BRM cannot substitute for BRG1 function, as our BRG1-KO cells express BRM and still show a strong DSB repair defect and all the previously discussed HR and DDR defects (Hays et al., 2020). It is possible that BRM can have overlapping functions with BRG1 and thus can compensate for some functions and not others, but the real function of BRM in DSB repair still is unclear and needs to be addressed in future studies.

### AT-rich interacting domain-containing subunits: ARID1A, ARID1B, and ARID2

**FIGURE 4:**
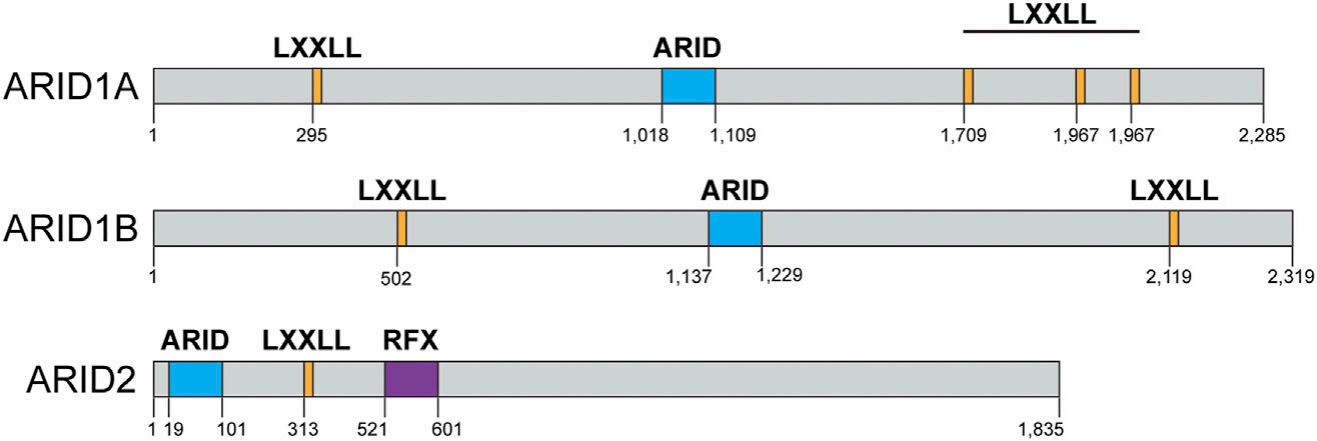
AT rich interacting domain (ARID)-containing proteins domain structures: ARID1A, ARID1B, ARID2. ARID1A contains four LXXLL domains (orange) and an ARID domain (blue). ARID1B contains two LXXLL domains (orange) and one ARID domain (blue) and ARID2 contains one ARID domain (blue), one LXXLL domain (orange), and a regulatory factor binding to the X-box (RFX, purple) DNA binding domain. Domain structure information obtained from NCBI and cBioPortal.

Two of the three SWI/SNF complexes in somatic mammalian cells contain one ARID domain-containing subunit (Figure 1). The cBAF complex can contain either ARID1A or ARID1B, while the PBAF complex contains exclusively ARID2 (Mashtalir et al., 2018). There is no ARID domain-containing subunit in the ncBAF complex and the functional implications for this peculiarity are unknown (Mashtalir et al., 2018; Michel et al., 2018). ARID1A is the most frequently mutated subunit within the SWI/SNF complex in human cancers (Figure 2) (Kadoch et al., 2013). ARID1A is mutated in ovarian clear cell, endometroid, and uterine endometroid carcinomas, among other cancer types (Jones et al., 2010; Guan et al., 2011; Caumanns et al., 2018; Xu and Tang, 2021). Mutations can be found all along the gene, most are non-sense mutations that result in truncated proteins. At the protein level, these subunits do not contain much more than LXXLL domains, an ARID domain, and ARID2 contains another DNA binding domain (Figure 4). Once again, most of the work regarding ARID1A focuses on its function in transcription, but several studies have shown that this subunit is also important for the repair of DSBs, thus ARID1A-mutated cancers likely have a DNA repair vulnerability that can be exploited therapeutically.

### The role of ARID1A in DSB repair

The Peng lab showed that ARID1A (also known as BAF250A or p270) interacts with the ATR kinase and is recruited to DSBs through this interaction (Shen et al., 2015). This ARID1A interaction with ATR was mapped to its C terminal domain, which agrees with more recent work suggesting that the N terminal domain of ARID1A likely interacts with DNA and not with other proteins or components of the SWI/SNF complex (Mashtalir et al., 2018). This study showed that inactivation of ARID1A results in a defect in both HR and single-strand annealing (SSA) repair. Both of these repair mechanisms require DNA end resection and the authors proposed that ARID1A stimulates DNA end resection (Shen et al., 2015). In agreement with a defect in DNA end resection, they showed that inactivation of ARID1A results in a defect in the activation of ATR and in the activation of the G2/M checkpoint. Importantly, they showed normal ATM activation in cells lacking ARID1A, as we showed for cells lacking BRG1 (Shen et al., 2015; Hays et al., 2020). They also observed a reduction in nucleosome density at DSBs upon the induction of the break in control cells and an increase in nucleosome density at DSBs in cells lacking ARID1A and proposed that this chromatin remodeling event somehow stimulates DNA end resection (Shen et al., 2015). We and others have also observed this reduction on nucleosome density at DSBs in control cells and an increase in nucleosome density at break sites in cells lacking BRG1, in addition to the defect in DNA end resection (Velez-Cruz et al., 2016; Wiest et al., 2017; Chen et al., 2019; Hays et al., 2020). Importantly, they also showed that inactivation of ARID1A renders cells sensitive to PARPi in cell culture and in mice xenografts. This study shows a clear function for ARID1A in the repair of DSBs and specifically stimulating ATR activation and DNA end resection. Taking into consideration the fact that ARID1A does not have any catalytic activity, the most likely possibility is that this subunit is responsible for the recruitment of a BRG1-containing cBAF complex to DSBs. Thus, ARID1A is likely required for the BRG1 function in DNA end resection, although this has not been shown directly yet. It is worth noting that in multiple studies regarding the function of ARID1A in transcription using ChIP-Seq (chromatin immunoprecipitation coupled to next-generation sequencing) the loss of ARID1A results in the loss (to a large extent) of SWI/SNF chromatin remodeling complexes binding to gene promoters and enhancers, thus suggesting that ARID1A does play a role in the recruitment of these complexes to genomic loci (Mathur et al., 2017; Wang et al., 2020).

Another study also identified cells lacking ARID1A as sensitive to IR and PARPi (Park et al., 2019). Park et al. (2019), however, showed that inactivation of ARID1A results in the attenuation of NHEJ and Alt-NHEJ, with some reduction in HR. The authors showed that the defect in NHEJ stems from the delayed recruitment of the 53BP1-RIF1 protein complex to DSBs in cells lacking ARID1A. Importantly, they also showed that cells lacking ARID1A displayed reduced chromatin accessibility near enzyme-induced DSBs, which is similar to the increased nucleosome density at DSBs reported by us and others in cells lacking BRG1 or ARID1A (Shen et al., 2015; Velez-Cruz et al., 2016; Chen et al., 2019; Hays et al., 2020). Park et al. (2019) also showed that cells lacking ARID1A are sensitive to PARPi *in vitro* and *in vivo* using mouse xenografts and that the toxicity of PARPi can be increased when combined with low dose IR. Although at least two studies have shown that the inactivation of ARID1A renders cells sensitive to PARPi, there is one recent genetic screen study that identified ARID1A as a gene that, when mutated, conferred resistance to PARPi and crosslinking agents such as cisplatin (Hu et al., 2018). These differences may be related to the different cell lines used in different studies (HCT116 for Shen et al., 2015 vs. MCF10A for Hu et al., 2018, respectively).

### The role of ARID1B in DSB repair

An earlier study by Watanabe, R. et al. also showed a defect in NHEJ upon the inactivation of either ARID1A or ARID1B (Watanabe et al., 2014). They proposed that the cBAF complex is important for the recruitment of the KU70/80 to DSBs and thus NHEJ. Using siRNAs against various SWI/SNF subunits and analyzing the recruitment of GFP-KU to laser microirradiation sites, they showed that either ATPase (BRG1 or BRM), ARID1A, ARID1B, SNF5, BAF60A, BAF60C, BAF155, and BAF170 are all required for the recruitment of GFP-KU70/80 to laser-induced DSBs and thus NHEJ (Watanabe et al., 2014). Downregulation of these SWI/SNF subunits also render cells somewhat sensitive to IR in this study and the authors showed that there was a subunit stability dependency between ARID1A, BAF155, BAF170, and SNF5. One caveat of this study is that the authors used H1299 non-small cell lung cancer cells, which do not express BRG1, and thus the effect that the inactivation of ARID1A or ARID1B may have in repair should be viewed as in addition to the lack of BRG1. Given that ARID1A and ARID1B do not have any catalytic activity, and the current view of their function is to recruit the SWI/SNF catalytic activity to a particular genomic locus and if this cell line only expresses BRM and not BRG1, the function of ARID1A and ARID1B may have been already impaired, as there is no BRG1 to be recruited. In the absence of BRG1, ARID1A and ARID1B can only recruit BRM to any genomic locus and studies suggest that BRM plays a minor role in repair (Reyes et al., 1998; Velez-Cruz et al., 2016; Hays et al., 2020).

ARID1B can also be found in the cBAF complex, although it is mutually exclusive with ARID1A (Mashtalir et al., 2018). ARID1B shares 60% sequence identity with ARID1A, also contains an ARID domain (Figure 4), and is also mutated (although at lower frequency) in a variety of cancers such as basal cell carcinoma, melanoma, and some gynecological cancers (Figure 2) (Kadoch et al., 2013; Mashtalir et al., 2018). Since the cBAF complex requires either ARID1A or ARID1B, the inactivation of ARID1B has been viewed as a potential vulnerability for ARID1A-mutated cancers (Helming et al., 2014). While ARID1A has garnered most of the attention, studies have established that while the lack of ARID1B by itself may not result in a pronounced repair defect, ARID1B can be targeted in cells that lack ARID1A. Indeed, the targeting of ARID1B in cells lacking ARID1A results in the inactivation of the cBAF complex, as one of these two subunits is required for the assembly of cBAF (Mashtalir et al., 2018). Inactivation of ARID1A and ARID1B simultaneously results in the near complete loss of SWI/SNF complexes binding to chromatin in the context of transcription, even affecting the distribution of PBAF complexes, which do not contain either subunit (Wang et al., 2020). In the context of DNA repair, there are no studies describing the function of ARID1B or BRM. There is a study showing that inactivation of ARID1B in colorectal cancer cells that do not express ARID1A can further sensitize these cells to IR (Niedermaier et al., 2019). Using a panel of colorectal cancer cells that express ARID1A vs. a panel of ARID1A-deficient cells, the authors showed that downregulation of ARID1B using siRNA increased the sensitivity to IR in cells lacking both subunits. The increased sensitivity was modest, but the authors also showed that this sensitivity was due to a defect in HR in these cells, as RAD51 foci formation was impaired in cells lacking both subunits (Niedermaier et al., 2019).

### The role of ARID2 in DSB repair

ARID2 (also known as BAF200) is the sole ARID-containing subunit of the PBAF complex (Figure 1) (Mashtalir et al., 2018). Once again, most of the studies related to this subunit are focused on its role in transcription of various programs related to carcinogenesis, differentiation, development, and cell growth (Xu et al., 2012; Savas and Skardasi, 2018; Wang et al., 2020). Elegant biochemical work from the Kadoch lab regarding the modular assembly of SWI/SNF complexes showed that inactivation of ARID2 impairs the assembly of the PBAF complex beyond its core components (Mashtalir et al., 2018). There is one study by the Pezza lab describing a novel function for ARID2 in HR (Castro et al., 2017). The authors showed that downregulation of ARID2 by siRNA in U2OS cells resulted in slower γH2AX clearance after etoposide treatment, also cells lacking ARID2 were sensitive to etoposide and IR. The increased sensitivity and slower γH2AX clearance were comparable to that observed upon downregulation of BRG1 through siRNA (Castro et al., 2017). Using a reporter system, they showed that downregulation of ARID2, BAF180 (also known as PBRM1), and BRG1 impaired HR, but downregulation of ARID1A did not. These results are in conflict with multiple studies showing that inactivation of ARID1A impairs HR, as discussed above. Using chromatin immunoprecipitation (ChIP) and restriction enzyme-mediated breaks they showed that downregulation of either ARID2 or BRG1 impaired the recruitment of RAD51 to DSBs. They also showed that ARID2 interacts with RAD51 independent of BRG1 and proposed that ARID2 was important for the removal of RPA from the ssDNA generated during DNA end resection and for the recruitment of RAD51. The authors also showed that ARID2 can form a complex with BAF180 that is independent of BRG1, and this complex may be responsible for this novel function in repair. The authors did not assess the activation of the DDR kinases (ATM or ATR), as has been done for ARID1A. In summary, this work identifies a role for ARID2 in HR, through the removal of RPA for the formation of RAD51 filaments. The authors states, however, that this novel function for ARID2 may not require BRG1 (Castro et al., 2017).

The presumptive function for the ARID components of the SWI/SNF complexes is the recruitment of these complexes to various genomic loci, yet most of the DNA repair studies that describe their role fail to test this function. None of the studies described here tested whether the repair defect observed upon the downregulation of any of the ARID subunits is worsened upon downregulation of BRG1 or BRM, or whether the recruitment of BRG1 or BRM to DSBs is affected by the inactivation of ARID1A or ARID1B. These details are still important because they answer the most basic question regarding the function of these subunits within the SWI/SNF complexes. Based on the studies described here we can conclude that ARID1A is important for the repair of DSBs through HR and its function is likely the recruitment of the BRG1 ATPase, while the latter has not been shown directly. The function of ARID1B and ARID2 in DNA repair, on the other hand, still requires more in-depth analysis that would resolve the conflicting results of the published work discussed here.

### Bromodomain-containing subunits: PBRM1, BRD7, BRD9

Bromodomains are protein domains that bind to acetylated lysine residues and are generally thought of as important epigenetic “readers” as they “read” the epigenetic code when they bind to genomic regions that contain certain histone acetylation marks (Müller et al., 2011; Papavassiliou and Papavassiliou, 2014). The SWI/SNF complexes have five subunits that contain bromodomains: BRG1, BRM, PBRM1, BRD7, and BRD9 (Figure 3), which means that all three SWI/SNF complexes have bromodomain-containing subunits. In the context of transcription regulation bromodomains are thought to help in the recruitment of SWI/SNF complexes to specific genomic regions, although it is unclear why these complexes would need so much redundancy in terms of bromodomains. There are several small molecules known as bromodomain inhibitors (BDi) that can block these domains and can be helpful for identifying the function of these subunits. BDi have also the potential to become therapeutic for various diseases (Delmore et al., 2011; Floyd et al., 2013; Shu et al., 2020). The function of these bromodomain-containing proteins in DNA repair is even less clear than within the context of transcription regulation.

### The role of BRG1 and BRM bromodomains in DSB repair

First, both ATPase subunits contain bromodomains in their C terminals (Figure 3) (Vangamudi et al., 2015). The function(s) of these bromodomains on BRG1 and BRM is unclear. Studies have used small molecule bromodomain inhibitors to define the function of these domains with mixed success. One study used the expression of the BRG1 bromodomain dimer (BRDD) to test the effect it would have on the sensitivity of these cells to IR (Kwon et al., 2015a). The authors observed that overexpression of the BRG1 bromodomain monomer or BRDD displaces endogenous BRG1 from chromatin, which the authors called a dominant negative effect. Cells expressing BRDD showed a DNA repair defect after IR (as observed through the comet assay), impaired γH2AX foci formation, impaired 53BP1 foci formation, and increased sensitivity to IR, etoposide, and doxorubicin. Using xenografts in nude mice they also showed that mice with tumors expressing BRDD and treated with IR developed smaller tumors than mice expressing a vector control, thus suggesting that the sensitization to DNA damage also occurs *in vivo*. Interestingly, overexpression of BRDD had no effect on the activation of the ATM or ATR kinases or the induction of senescence after IR (Kwon et al., 2015a). It is surprising that the expression of the BRDD alone seems to remove endogenous BRG1 and likely SWI/SNF complexes from chromatin as SWI/SNF complexes themselves have more bromodomain-containing proteins. Whether the expression of BRDD also removes complexes containing BRM is still unknown, as the authors did not comment on that possibility. It would also be of interest to determine what effect the expression of BRDD would have on the transcriptional function of these complexes. Another study used a small molecule inhibitor of the BRG1/BRM bromodomain, PFI-3 (Vangamudi et al., 2015). The authors showed treatment with PFI-3 can displace an ectopically-expressed BRM bromodomain, but not full length BRM or the SWI/SNF complex. Moreover, in cells lacking BRG1 that depend on BRM for growth, PFI-3 had no effect in cell proliferation, thus suggesting that the bromodomain of BRM is dispensable for the oncogenic function of SWI/SNF complexes in lung cancer. Indeed, the authors concluded that the ATPase domain of BRM and BRG1 are required for their oncogenic activity, while their bromodomains are not. The lack of anti-proliferative activity for PFI-3 argues for the other bromodomain subunits of SWI/SNF complexes serving a backup function and thus a reason for redundancy in terms of bromodomains and SWI/SNF complexes. Future studies should try to mutate or delete the bromodomain in BRG1 to study its role (if any) in DSB repair.

### The role of PBRM1 in DSB repair

**FIGURE 5:**
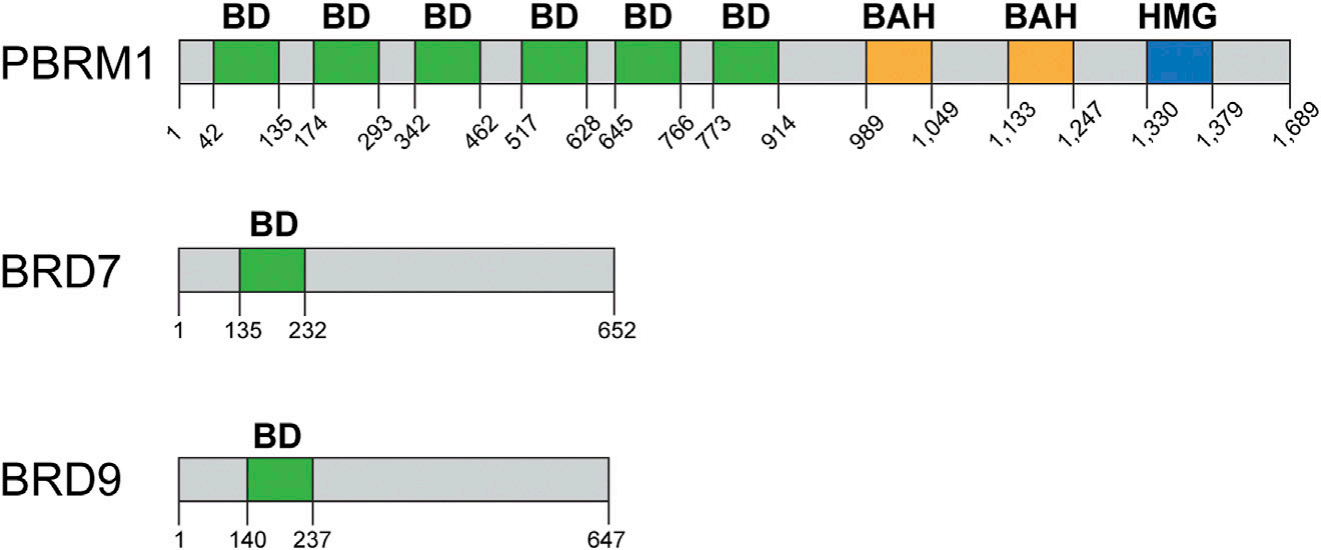
Domain structure of bromodomain-containing proteins: PBRM1, BRD7, BRD9. PBRM1 (BAF180) contains six bromodomains (green), two bromo-adjacent homology domains (BAH, yellow), and one high mobility group (HMG, blue) DNA binding domain. BRD7 and BRD9 contain one bromodomain each (green). Domain structure information obtained from NCBI and cBioPortal.

The PBRM1 (BAF180) subunit of the PBAF complex contains six bromodomains, two bromo adjacent homology (BAH) domains, and one high mobility group (HMG) domain (Figure 5) and its inactivation has been linked to a DSB repair defect. PBRM1 is mutated at high rates in renal clear cell carcinomas, among other cancers (Figure 2). The Downs lab showed that PBRM1 is important for the transcriptional repression that occurs near DSBs, which is important for proper DSB repair (Shanbhag et al., 2010; Kakarougkas et al., 2014). The authors used a system designed by the Greenberg lab to monitor the ATM-dependent transcriptional repression that occurs near a DSB (Shanbhag et al., 2010). The authors showed that downregulation of BRG1 or PBRM1 impairs the transcriptional repression that occurs near DSBs in control cells. Interestingly, this repression also requires the ATPase activity of BRG1 and cannot be mediated by BRM, as downregulation of BRM did not impair transcriptional repression (Kakarougkas et al., 2014). Transcriptional repression near DSBs seems to be a mechanism to prevent conflicts between transcription and repair machineries. The authors also showed that downregulation of PBRM1, BRG1, or expression of an ATPase-dead BRG1 resulted in higher levels of γH2AX foci, thus suggesting a DSB repair defect. This DSB repair defect was only detectable early after IR and likely mediated by the NHEJ pathway, as at 24 h post IR γH2AX foci were resolved in control cells and in cells lacking either BRG1 or PBRM1. The higher levels of γH2AX observed by the authors is in agreement with our studies (even though we observed sustained higher levels of γH2AX in the absence of BRG1 at all time points) but is in conflict with earlier studies showing that BRG1 is required for γH2AX foci formation (Park et al., 2006; Lee et al., 2010; Hays et al., 2020). The authors propose that the delay in the repair of DSBs is due to the defect in transcriptional repression near DSBs, as the delay in repair can be overcome by inhibiting transcription globally (Kakarougkas et al., 2014). At the chromatin level, the transcriptional repression near DSBs required ubiquitylation of histone H2A at lysine 119 (H2A-K119Ub), which is mediated by the Polycomb repressive complex 1 (PRC1) and requires PBRM1. The Polycomb repressive complex 2 (PRC2) and its EZH2 subunit, which mediates H3K27me3, were also involved in the transcriptional silencing nearby DSBs. Downregulation of either PRC1 or PRC2 abolished the transcriptional silencing induced by DSBs. The authors did not assess the chromatin state at the DSB or at the promoter level of the repressed reporter to determine how exactly was PRBM1 or SWI/SNF contributing to the repression. Importantly, the authors showed that PBRM1 mutations found in cancers failed to repress transcription nearby DSBs, thus suggesting that this function for PBRM1 may be associated with carcinogenesis or cancer progression.

### The role of BRD7 in DSB repair

BRD7 is another bromodomain-containing subunit of the SWI/SNF complexes, and it is part of the PBAF complex specifically (Figure 1; Figure 5) (Yu et al., 2016). BRD7 is associated with various types of cancer including melanoma, endometrial, and bladder cancer (Figure 2), and it has mostly been studied in the context of transcription and its association with p53 (Burrows et al., 2010; Yu et al., 2016). There are reasons to believe that BRD7 may play a role in DNA repair. For instance, inactivation of BRD7 in mice results in male mice that are sterile due to defects during spermatogenesis, which also occurs in testis-specific BRG1-KO mice (Kim et al., 2012; Wang et al., 2012, 2016). These sperm development defects are typical of mice with defects in meiotic recombination, which is similar to homologous recombination repair.

A recent study investigated the role of BRD7 in the transcriptional repression that occurs near DSBs (Hu et al., 2020). Using the same system described above designed by the Greenberg lab, the authors showed that BRD7, PBRM1, BRG1, and ARID2 (all part of the PBAF complex, Figure 1) are required for transcriptional repression near DSBs. The authors also showed that the ncBAF subunits BRD9 and GLTSCR1 also are important for transcriptional repression near DSBs, while downregulation of BAF47 and BAF57 had no effect, and downregulation of ARID1A had an intermediate effect in repression that was not explored any further (Hu et al., 2020). They also showed that, in addition to ATM, inhibition of DNA-PK and PARP also impaired transcriptional repression near DSBs. Importantly, the authors showed that this transcriptional repression near DSBs is mediated by the PRC2 and the nucleosome remodeling and deacetylase (NuRD) complexes, in addition to the previously described PRC1 complex. The authors also identified and characterized a defect in HR in cells lacking BRD7. They observed signs of a defect in DNA end resection that stemmed from their proposed function for BRD7 in the recruitment of the MRN complex to DSBs, as they also showed that BRD7 is in a complex with the MRN complex. In agreement with a defect in MRN recruitment, they showed a defect in ATM activation after DNA damage (CPT or IR) and a defect in HR. The authors also showed that inactivation of BRD7 sensitizes cells to CPT, IR, and PARPi. They also showed ATM and ATR phosphorylate BRD7 after DNA damage and these phosphorylation events are important for interactions between various repair proteins and BRD7 (Hu et al., 2020).

At this time, it is difficult to say whether the bromodomains contained within BRG1 or BRM play a role in DSB repair, as these domains have not yet been deleted or mutated to study their effect on DSB repair, and studies using small molecule inhibitors suggest that the ATPase domain is more important than the bromodomain for cell proliferation (Vangamudi et al., 2015). Future studies that focus on these bromodomains will further stimulate the development of small molecule inhibitors that may in the future be used to sensitize cells to DNA damage or enhance the effects of radiotherapy or chemotherapy. It is also important to promote the development of better BDi that are more effective and selective in order to be able to definitely identify the function of these protein domains.

### Therapeutic opportunities by targeting SWI/SNF complexes in cancer

Radiotherapy and the vast majority of chemotherapeutic agents kill cancer cells by inducing overwhelming amounts of DNA damage, including DSBs (Jackson and Helleday, 2016; Nickoloff et al., 2017). Since multiple subunits of the SWI/SNF chromatin remodeling complexes are mutated at high frequencies in various cancers, identifying SWI/SNF subunits that are important for the DNA repair function of these complexes also identifies DNA repair vulnerabilities that can be exploited therapeutically for the treatment of SWI/SNF-mutated cancers. This becomes particularly important when we take into consideration the fact that these complexes are known to play a role in HR and HR-deficient cancers are sensitive to DSB-inducing agents and PARPi (Helleday, 2010; Benafif and Hall, 2015; Dréan et al., 2016; Jackson and Helleday, 2016; Nickoloff et al., 2017).

Virtually every study that has investigated the role of BRG1 in DNA repair has reported that inactivation of BRG1 renders cells sensitive to DNA damage by IR, or etoposide, or other DSB-inducing drugs, in agreement with this ATPase being important for the repair of DSBs (Park et al., 2006; Lee et al., 2010; Kwon et al., 2015a; Qi et al., 2015; Smith-Roe et al., 2015; Wu et al., 2016; Chen et al., 2019; Hays et al., 2020). Moreover, a recent CRISPR/Cas9 screen also identified BRG1 (*SMARCA4* gene in humans), ARID2, and PBRM1 as sensitive to etoposide (Olivieri et al., 2020). This screen did not identify all cBAF components or sensitivity to other forms of DNA damage likely due to the cell line used (RPE-1). At this time we can say that the development of ATPase inhibitors would likely be more useful in terms of cancer therapy (Vangamudi et al., 2015; Wu et al., 2016). While there are not any studies describing the role of BRM in DSB repair, a study showed that inactivation or downregulation of BRM in BRG1-mutated lung cancer cells further sensitizes these cells to IR (Zernickel et al., 2019). BRM has also been identified as essential in BRG1-mutated cell lines in various studies (Oike et al., 2013; Hoffman et al., 2014; Rago et al., 2020). This apparent synthetic lethal (SL) interaction between BRG1 and BRM seems to be cell line-specific, as there are various cell lines that do not express either ATPase but still survive (*e.g*., SW13 adrenal adenocarcinoma cells). Interestingly, multiple studies, including ours, have shown that inactivation of BRG1 does not result in sensitivity to PARPi (Gupta et al., 2020; Hays et al., 2020). It is unclear why HR-deficient cells, such as those that do not express BRG1, are not sensitive to PARPi. One potential explanation is that in the absence of BRG1, BRM is still present and providing a minimal amount of HR thus conferring resistance to PARPi. Indeed, one study showed that simultaneous downregulation of both ATPases, BRG1 and BRM, results in sensitivity to PARPi (Smith-Roe et al., 2015). There is also the possibility that inactivation of BRG1 in different cell lines may yield differences in sensitivity to PARPi, since we know that different cell lines may have different mutations in various signaling pathways that may affect the outcome of the downregulation or inactivation of BRG1, BRM, or both.

In addition to the ATPases, the ARID-containing subunits have been shown to be important for the sensitivity of cells to DSB-inducing agents. The cBAF complex can have either ARID1A or ARID1B at a given time, while the PBAF complex contains ARID2 (Mashtalir et al., 2018) (Figure 1). ARID1A is the most frequently mutated subunit within the SWI/SNF complex and up to 50% of ovarian clear cell carcinomas are mutated on this subunit (Kadoch et al., 2013; Caumanns et al., 2018). A similar SL interaction to that between BRG1 and BRM has been shown for ARID1A-mutated ovarian cancer cells, which are then dependent on ARID1B (Helming et al., 2014). A similar study was also published using lung cancer cell lines (Watanabe et al., 2014). A study in colorectal cancer cells showed that inactivation of ARID1B in ARID1A-mutated cells increases their sensitivity to IR, though only modestly (Niedermaier et al., 2019). Another study showed that ARID1A-deficient endometrial cells can become sensitized to IR upon treatment with PARPi (Park et al., 2019). ARID1A-deficient cells are also sensitive to ATR inhibitors (ATRi), which have potential for cancer therapy in the near future (Shen et al., 2015; Williamson et al., 2016; Yazinski and Zou, 2016; Hopkins et al., 2022). These studies show that ARID1A has the potential to become an effective target for cancer therapies. Moreover, studies also suggest that ARID1B can be a particularly interesting target in ARID1A-mutated cancers. Regarding ARID2, more studies will have to be performed in order to clarify the discrepancies that exist today with respect to its potential function in HR and the silencing of transcription nearby DSBs (Castro et al., 2017; Hu et al., 2020).

Bromodomain-containing subunits have also been shown to be important for DSB repair. BRD7 inactivation results in a modest increase in sensitivity to CPT and PARPi (Hu et al., 2020). BRD7 is mutated or downregulated in various cancers including bladder, endometrial, hepatobiliary, melanoma, and others (Yu et al., 2016). The sensitization of cells lacking these BD-containing subunits to DNA damage argues for the development of better and more selective BDi for potentially enhancing the effects of chemotherapies or radiotherapy.

Finally, it is important to pay more attention to the cell lines and altered signaling pathways context in the studies describing the effects that downregulation or inactivation of a SWI/SNF subunits are performed in. These differences may explain some discrepancies regarding the sensitivity of certain cells lacking a particular subunit to certain drugs such as PARPi. These differences can also identify signaling pathways that when mutated, result in a SL interaction with SWI/SNF mutations. For instance, loss of the *PTEN* phosphatase tumor suppressor sensitizes prostate cancer cells to BRG1 downregulation (Ding et al., 2019). Similarly, inhibition of the EZH2 methyltransferase subunit of the PRC2 complex in ARID1A-mutated ovarian cancer cells also results in a SL interaction related to PI3 kinase-AKT signaling (Bitler et al., 2015). While these SL interactions may be unrelated to the role of SWI/SNF in DNA repair, they may be very important for the advancement of therapies against SWI/SNF-mutated cancers.

## Conclusion

The importance of SWI/SNF complexes in carcinogenesis through their role in transcription is well established. The more recently uncovered role of the SWI/SNF complexes in the repair of DSBs is now well established also (Hohmann and Vakoc, 2014; Arnaud et al., 2018; Valencia and Kadoch, 2019). The next step is to try to use the information we have gathered regarding these complexes in the repair of DSBs to improve the efficacy of cancer therapies. More studies will have to be performed in order to answer lingering questions regarding these complexes, but these studies will also stimulate the development of more and better tools to improve our studies and their impact in the treatment of SWI/SNF-mutated cancers.

**FIGURE 6:**
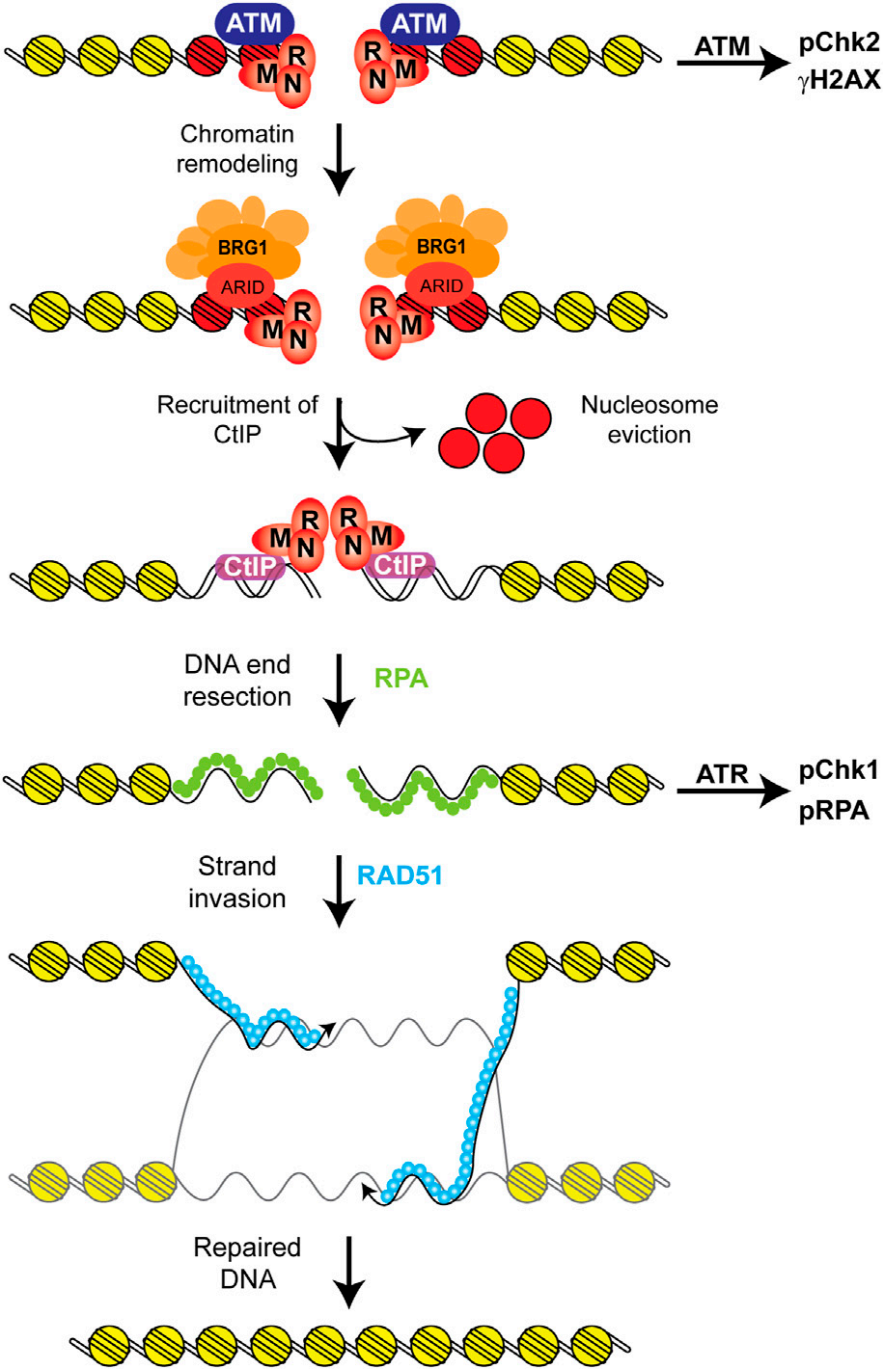
Proposed model for the role of SWI/SNF complexes in HR. After the recognition of the DSB by the MRN complex and the ATM kinase, DNA damage signaling is initiated by ATM and there is a chromatin remodeling step mediated by a BRG1-containing SWI/SNF complex that reduces nucleosome density at the DSB (Shen et al., 2015; Velez-Cruz et al., 2016; Chen et al., 2019; Hays et al., 2020). We propose that this step likely results in the eviction of these nucleosomes at the DSB, which likely contain γH2AX (red) and that this chromatin remodeling step stimulates DNA end resection by stimulating or stabilizing the recruitment of the CtIP nuclease to the break site. We also propose that the ARID1A subunit is likely responsible for anchoring the SWI/SNF complex at the DSB, as inactivation of this subunit results in very similar repair defects as those observed upon the inactivation of BRG1 (Shen et al., 2015; Hays et al., 2020). This model is in agreement with the fact that ATM signaling is not affected by the absence of BRG1, but ATR signaling is attenuated upon BRG1 inactivation due to the defect in DNA end resection. This model is also in agreement with work showing that nucleosomes block DNA end resection and that the processes of resection and the removal of nucleosomes are coupled (Mimitou et al., 2017; Wiest et al., 2017; Peritore et al., 2021).

First, the model for the function of SWI/SNF complexes in HR still requires more work. An earlier model proposed a function for BRG1 removing RPA from ssDNA and replacing it with RAD51 (Qi et al., 2015). A similar model was proposed for the function of ARID2, but this model does not require the ATPase activity of BRG1 or BRG1 itself, and in the case of ARID2 the authors proposed that ARID2 may be working together with BAF180 as a separate complex from the typical PBAF complex (Castro et al., 2017). There is more ample evidence from various groups, including ours, proposing a function for BRG1 stimulating DNA end resection and HR (Figure 6). We propose a model in which upon the detection of the DSB by the MRN complex and the ATM kinase, there is a chromatin remodeling step mediated by a BRG1-containing SWI/SNF complex (Figure 6). During this chromatin remodeling step, we and others observe a reduction in nucleosome density at the site of the break and we propose that this reduction in nucleosome density promotes or stimulates DNA end resection (Shen et al., 2015; Velez-Cruz et al., 2016; Chen et al., 2019; Hays et al., 2020). We, indeed, observe that in the absence of BRG1 the recruitment of CtIP is impaired thus explaining the defect in resection (Hays et al., 2020). This defect in DNA end resection explains the impaired activation of the ATR kinase in the absence of BRG1 (Shen et al., 2015; Smith-Roe et al., 2015; Hays et al., 2020). This model is also in agreement with work in yeast related to DNA end resection that has shown that SWI/SNF complexes are important for the resection step and for the removal of nucleosomes and that these two processes are coupled (Mimitou et al., 2017; Wiest et al., 2017; Peritore et al., 2021). There are still some questions regarding our model. For instance, we propose that BRG1 likely evicts nucleosomes at the site of the break, but that has not yet been shown. We also propose that this change in chromatin structure at DSBs affects CtIP recruitment or its retention at DSBs (Hays et al., 2020). Two previous studies showed that the p400 ATPase was required for the eviction of γH2AX nucleosomes from the break sites and the incorporation of the histone variant H2AZ (Xu et al., 2010; Xu et al., 2012 Y.). These studies also showed that the incorporation of the histone variant H2AZ at DSBs reduced DNA end resection and the authors suggested that H2AZ may affect CtIP activity by demarking chromatin boundaries to limit resection. Thus, it is possible that the chromatin environment at DSBs can modulate the activity of CtIP. We also propose that an ARID or a bromodomain-containing subunit is likely responsible for the anchoring of the SWI/SNF complex to DSBs. Based on work regarding the role of SWI/SNF complexes in transcription, we believe that ARID1A is likely that subunit, as inactivation of ARID1A results in an HR defect and an increase in nucleosome density at DSBs, similar to that observed in the absence of BRG1 (Shen et al., 2015; Chen et al., 2019; Hays et al., 2020). A similar defect in DNA end resection was observed in the absence of BRD7, thus it is possible that this subunit may also be responsible for the anchoring of the BRG1-containing SWI/SNF complex to DSBs (Hu et al., 2020).

One interesting question regarding the role of SWI/SNF complexes in DSB repair is which complex mediates which function. Initially it seemed clear that cBAF may perform a role in HR and the PBAF complex would be involved in the silencing of transcription nearby DSBs, but the finding that BRD7 and ARID2 may also play a role in HR somewhat refutes this model. This function of BRD7 in HR notwithstanding, BRG1, PBRM1, ARID2, and BRD7 are all subunits within the PBAF complex and are all important for the silencing of transcription nearby DSBs, while BRG1 and ARID1A are known to play a role in HR (Kakarougkas et al., 2014; Shen et al., 2015; Chen et al., 2019; Hays et al., 2020; Hu et al., 2020). Further studies will have to be performed to determine whether different SWI/SNF complexes perform different functions at DSBs.

Another interesting question relates to the process of DNA end resection. This step is critical for HR and several other ATPases are known to be recruited to DSBs and modulate this process. INO80 is a member of another family of ATPases important for the movement, eviction, and incorporation of histones into DNA. This complex was also shown to stimulate DNA end resection (Gospodinov et al., 2011). The p400 ATPase is also involved in resection, as discussed above (Xu et al., 2010; Xu et al., 2012 Y.). Thus, in addition to cBAF and PBAF modulating DNA end resection, there is INO80 and p400. The redundancy of ATPases is interesting, as most of them stimulate resection (with the exception of p400 which restrains resection). Why would we need multiple ATPases for the same process is an interesting question. These ATPases cannot possibly be performing the same function, as the inactivation of any of them results in a defect in resection. Future studies should address the interplay between these different complexes in order to further define the role of these chromatin remodelers in the resection process.

Finally, these studies argue for the development of new and better tools to further study the functions of SWI/SNF chromatin remodelers. While there are some SWI/SNF BDi available, better and more selective small molecules can improve the feasibility of cellular studies and preclinical work. Moreover, the development of small molecules that can selectively inhibit the ATPase domain of these complexes would also be of great use. Also, the development of small molecules that can disrupt the assembly of these complexes would greatly help further these studies, as has been done for the PRC2 complex (Kim et al., 2013). Studies related to the function(s) of SWI/SNF complexes in the preservation of genome integrity continue. Recent reports show that these complexes are also important for the removal of R loops and avoidance of replication-transcription machinery conflicts from the genome, which also preserve genome integrity (Bayona-Feliu et al., 2021; Tsai et al., 2021). The question still remains: how can we use the information regarding the function of these complexes in DNA repair to advance the treatments of SWI/SNF-mutated cancers?
