# Supplementary material for: The role of SWI/SNF chromatin remodelers in the repair of DNA double strand breaks and cancer therapy
Source: Front Cell Dev Biol. 2022 Dec 20;10:1071786. doi: 10.3389/fcell.2022.1071786 (PMC9810387; doi:10.3389/fcell.2022.1071786)
Supplement: Supplementary file 1 [file DataSheet1.PDF]

Supplementary Table 1: SWI/SNF subunits mutated in cancer. Data obtained from the TCGA through cBioportal showing the frequency of mutations in SWI/SNF subunits across different cancer types. The asterisk (*) denotes mutation data when the majority of mutations are amplifications, otherwise the majority of alterations are missense mutations or deletions. The TCGA data set contains 10,967 samples. Only the top four cancer types are shown.

**Complex**	**Subunit type**	**Subunit (Gene name)**	**Cancer type**
cBAF, PBAF, ncBAF	Catalytic	BRG1 (SMARCA4)	Endometrial Carcer (14.33%)<br>Melanoma (10.36%)<br>Ovarian epithelial tumor (9.76%)*<br>Esophagogastric cancer (8.52%)
cBAF, PBAF, ncBAF	Catalytic	BRM (SMARCA2)	Endometrial cancer (11.09%)<br>Bladder carcer (9.49%)<br>Esophagogastric cancer (9.32%)<br>Melanoma (8.78%)
cBAF, PBAF	Core	BAF47 (SMARCB1)	Endometrial cancer (4.27%)<br>Bladder cancer (3.65%)<br>Esophagogastric cancer (3.38%)<br>Colorectal cancer (2.36%)
cBAF, PBAF, ncBAF	Core	BAF155 (SMARCC1)	Endometrial cancer (7%)<br>Mature B-cell neoplasms (6.25%)*<br>Melanoma (6.08%)<br>Bladder cancer (3.65%)
cBAF, PBAF	Core	BAF170 (SMARCC2)	Endometrial cancer (9.9%)<br>Melanoma (7.66%)<br>Bladder cancer (6.08%)<br>Esophagogastric cancer (5.14%)
cBAF, PBAF	Core	BAF57 (SMARCE1)	Esophagogastric cancer (9.32%)*<br>Adrenocortical carcinoma (4.4%)<br>Endometrial cancer (4.27%)<br>Breast cancer (4.24%)*
cBAF, PBAF, ncBAF	Core	BAF60A (SMARCD1)	Adrenocortical carcinoma (4.4%)*<br>Mature B-cell neoplasms (4.17%)*<br>Endometrial cancer (4.1%)<br>Melanoma (2.93%)
cBAF, PBAF, ncBAF	Core	BAF60B (SMARCD2)	Breast cancer (6.64%)*<br>Pleural mesothelioma (5.75%)*<br>Endometrial cancer (4.78%)<br>Esophagogastric cancer (3.54%)
cBAF, PBAF, ncBAF	Core	BAF60C (SMARCD3)	Ovarian epithelial tumor (8.22%)*<br>Endometrial cancer (4.78%)<br>Adrenocortical carcinoma (4.4%)<br>Melanoma (4.05%)
cBAF, PBAF, ncBAF	Accessory	B actin (ACTB)	Mature B-cell neoplasms (8.33%)<br>Bladder cancer (8.03%)<br>Adrenocortical carcinoma (5.49%)*<br>Esophagogastric cancer (5.31%)*

cBAF,<br>PBAF,<br>ncBAF	Accessory	BAF53A<br>(ACTL6A)	Ovarian epithelial tumor (19.52%)*<br>Non-small cell lung cancer (19.28%)*<br>Head and neck cancer (15.87%)*<br>Cervical cancer (15.49%)*
cBAF,<br>PBAF,<br>ncBAF	Accessory	BAF53B<br>(ACTL6B)	Esophagogastric cancer (6.75%)*<br>Melanoma (5.63%)<br>Head and neck cancer (4.97%)*<br>Endometrial cancer (4.78%)
cBAF,<br>ncBAF	Accessory	SS18	Pancreatic cancer (5.98%)*<br>Sarcoma (5.1%)*<br>Ovarian epithelial tumor (4.45%)*<br>Endometrial cancer (4.27%)
cBAF,<br>ncBAF	Accessory	CREST<br>(SS18L1)	Colorectal cancer (8.08%)*<br>Ovarian epithelial tumor (6.68%)*<br>Sarcomas (5.49%)*<br>Endometrial cancer (5.12%)*
cBAF,<br>PBAF,<br>ncBAF	Accessory	BCL7A	Mature B-cell neoplasms (8.33%)<br>Endometrial cancer (2.39%)<br>Sarcoma (2.35%)*
cBAF,<br>PBAF,<br>ncBAF	Accessory	BCL7B	Non-seminomatous germ cell tumor (3.49%)*<br>Adrenocortical carcinoma (3.3%)*<br>Ovarian epithelial tumor (2.57%)*<br>Esophagogastric cancer (2.25%)*
cBAF,<br>PBAF,<br>ncBAF	Accessory	BCL7C	Endometrial cancer (5.12%)*<br>Breast cancer (4.24%)*<br>Melanoma (2.7%)<br>Esophagogastric cancer (2.25%)
cBAF	Accessory	BAF250A<br>(ARID1A)	Endometrial cancers (40.78%)<br>Bladder cancer (27.25%)<br>Esophagogastric cancer (21.7%)<br>Cholangiocarcinomas (13.89%)
cBAF	Accessory	BAF250B<br>(ARID1B)	Endometrial cancer (9.39%)<br>Melanoma (9.01%)<br>Cholangiocarcinomas (8.33%)<br>Bladder cancer (7.79%)
cBAF	Accessory	BAF45B<br>(DPF1)	Ovarian epithelial tumor (8.05%)*<br>Endometrial cancer (5.97%)*<br>Pancreatic cancer (5.43%)*<br>Bladder cancer (5.11%)*
cBAF	Accessory	BAF45C<br>(DPF3)	Endometrial cancer (3.24%)<br>Melanoma (2.93%)<br>Esophagogastric cancer (2.89%)<br>Cholangiocarcinomas (2.78%)
cBAF	Accessory	BAF45D<br>(DPF2)	Endometrial cancer (4.95%)<br>Head and neck cancer (4.78%)*<br>Melanoma (4.73%)<br>Bladder cancer (4.62%)*
PBAF	Accessory	BAF200<br>(ARID2)	Melanoma (17.57%)<br>Endometrial cancer (11.6%)<br>Bladder cancer (9%)

			Esophagogastric cancer (7.56%)
PBAF	Accessory	BAF180 (PBRM1)	Renal clear cell carcinoma (30.92%)<br>Cholangiocarcinomas (19.44%)<br>Endometrial Carcer (10.92%)<br>Melanoma (7.88%)
PBAF	Accessory	BRD7	Endometrial Carcer (5.63%)<br>Melanoma (4.95%)<br>Bladder cancer (3.65%)<br>Hepatobiliary cancer (2.96%)
PBAF	Accessory	BAF45A (PHF10)	Ocular melanoma (7.5%)<br>Cholangiocarcinomas (5.56%)<br>Endometrial cancer (4.78%)<br>Hepatobiliary cancer (3.49%)
ncBAF	Accessory	BRD9	Non-Small Cell lung cancer (13.87%)*<br>Adrenocortical carcinomas (13.19%)*<br>Bladder cancer (10.71%)*<br>Ovarian epithelial tumor (9.25%)*
ncBAF	Accessory	GLTSCR1 (BICRA)	Mature B cell neoplasms (6.25%)<br>Endometrial cancer (5.63%)<br>Cholangiocarcinomas (2.78%)<br>Colorectal cancer (2.69%)
ncBAF	Accessory	GLTSCR1L (BICRAL)	Esophagogastric cancer (8.84%)*<br>Melanoma (7.66%)<br>Mature B-cell neoplasm (6.25%)*<br>Endometrial cancer (5.63%)
